# Growth Performance and Carcass Traits Responses to Dried Distillers’ Grain with Solubles Feeding of Growing Awassi Ram Lambs

**DOI:** 10.3390/ani9110954

**Published:** 2019-11-12

**Authors:** Safa’ M. Hatamleh, Belal S. Obeidat

**Affiliations:** Department of Animal Production, Faculty of Agriculture, Jordan University of Science and Technology, Irbid 22110, Jordan; seef.islam.sh@gmail.com

**Keywords:** Awassi lambs, carcass traits, digestibility, DDGS, nutrient intake

## Abstract

**Simple Summary:**

Corn grain is considered to be the most important crop in the world. Corn dried distiller grains with solubles is a by-product of ethanol production. It is included at 0, 125, or 25 g/kg in lambs’ diets. This inclusion reduced the cost of diet, indicating the good possibility of using it in small ruminant diets.

**Abstract:**

This study considers the impact of dried distillers’ grain with solubles (DDGS) in diets of lambs. Randomly; 27 lambs were distributed to one of three diets. Diets were: a control diet (CON; *n* = 9), a 125 (DDGS125; *n* = 9) or a 250 g/kg DDGS (DDGS250; *n* = 9) of dietary dry matter (DM). The lambs were fed using these diets for 91 days. To assess carcass traits; five lambs were randomly selected at the end of the study. No significant differences were detected in intake and digestibility of DM; crude protein and fiber. Average daily gain did not differ among diets. Carcass characteristics did not differ among diets. With the exception of shear force and redness, which were greater in DDGS250 than in DDGS125 and CON diets, meat quality parameters were unaffected. Eye muscle area decreased in DDGS125 than in DDGS250 and CON diet. These results demonstrate that the feeding of lambs on DDGS at 125 or 250 g/kg DM did not have any impact on growth. These diets only had a simple effect on the characteristics of carcass and meat quality. These results suggest that it would be suitable to introduce these feeds into sheep nutrition in the future.

## 1. Introduction

Corn grain is considered to be the most important crop in the world. It is estimated that half of agricultural production of this grain is for non-nutritional purposes. Ethanol, produced from the corn, is commonly used as fuel for machines and hundreds of materials used in the medical and pharmaceutical industry. Corn dried distiller grains with solubles (DDGS) is a byproduct of ethanol produced from corn grain. The demand for this product is expected to increase due to an increase in conventional feeds cost and the increase of bio-fuel production in response to global environmental concerns. Furthermore, the use of DDGS in animal feed has increased significantly [1,2]. These residues account for 40% of the income of ethanol production plant. Livestock nutritionists emphasize the importance of maintaining preferential performance in efficiency and growth rate while keeping feed costs as low as possible [3]. Obeidat [2] reported that DDGS contains 273 g/kg crude protein (CP), where more than half of CP is considered bypass the rumen degradation, making it a good candidate to replace soybean meal (CP = 480 − 520 g/kg DM) as a feed product. 

Awassi sheep are a significant feature in the food chain in Jordan. It is one of the most important small ruminants that provide 28% of the locally produced milk and 62% of the red meat in a region where annual red meat consumption is estimated to be 10.8 kg per capita [4]. The population of small ruminants in Jordan is approximately 4.3 million head including sheep and goats [5]. Due to the shortage and availability of good quality forages and pastures, small ruminant husbandry has moved from nomadic grazing to semi-intensive and intensive husbandry, thus increasing the need to use cereal grains and protein sources in animal diets [4]. Based on this, feed cost has increased tremendously, which has led to reduced revenue and profit for farmers. Therefore, producers tend to use alternative feeds in ruminant diets to reduce feeding cost [6]. 

There is very little available data regarding the usage of DDGS in Awassi sheep feed. Recently, Obeidat [2] reported that growth performance, nutrient intake and digestibility was similar between growing lambs being fed DDGS at 0, 75 or 150 g/kg dry matter (DM). Similarly, another study reported that feeding DDGS to lambs at ratios of 0, 127 and 254 g/kg of dietary DM did not affect DM intake [7]. However, some literature has provided controversial results regarding carcass characteristics and meat quality [8,9]. Study the use and impacts of DDGS for sheep feeding in Jordan is limited at present. Therefore, this study will be an important contribution to the research and data pertaining to the performance growth and carcass traits of growing lambs. Aim of this research study was to evaluate the influence the feeding DDGS at 0, 125 or 250 g/kg on performance and carcass characteristics of growing lambs. 

## 2. Material and Methods

The Institutional Animal Care and Use Committee at Jordan University of Science and Technology (JUST) approved all procedures and methods used in this study (Protocol #: 16/3/3/410). 

This study was carried out using 27 male lambs (initial body weight (BW) = 15.3 ± 0.99 kg) in the Animal Farm of the Research and Training Department at the Faculty of Agriculture. Lambs were born and raised in the Animal Farm from the sheep herd at JUST. Lambs used in the study were weighed, ear tagged and had a health check. Lambs were randomly divided into three groups. The groups were fed different diets using diets containing DDGS at 0 (CON), 125 DDGS (DDGS125) or 250 g/kg DDGS (DDGS250) DM respectively (Table 1). These diets were fed to the lambs for 91 days. The first 7 days were used for adaptation and the remaining 84 days were utilized for data collection. Lambs were retained in individual pens (1.5 × 0.75 m). The feed was provided ad libitum daily at 08:00. Lambs had unimpeded access to water during the study. Dried distillers’ grain with solubles was purchased from a commercial feed mill then incorporated into the diet. Throughout the study, feed refusals were collected, weighed and stored (−20 °C) daily. Lamb BW was collected every two weeks to calculate feed efficiency (FCR; DM intake: gain). 

To estimate nutrient digestibility and N balance, six lambs were randomly chosen from each group of diet on the 56th day of the experiment and were placed in metabolic cages (1.05 × 0.80 m) suitable for collection of feces and urine. The animals were given five days to adapt followed by five days where their feces and urine were collected. All the methods used in this experiment and analysis of feces and urine samples replicated the procedures described in Obeidat et al. [10]. Chemical composition of the samples of diets, refusal and feces were analyzed as described in Alshdaifat and Obeidat [1]. 

On the last day of the study, five lambs were chosen randomly from each group to determine carcass characteristics and meat quality. Their feed was cut for 18 h and the lambs were weighed to find their final fasting weight. Animals were slaughtered by an experienced trainee using a standard slaughter procedure. After the slaughter of lambs, hot carcass weights were recorded. The weight of the heart, lungs and trachea, kidneys, spleen, liver, kidneys and mesenteric fat (non-carcass components) was recorded. The carcasses were then transferred to a refrigerator and kept at 4 °C for 24 h. On the second day, the weights of the cold carcasses were measured to calculate the dressing percentage. The fat tail was then cut, and its weight recorded. Cold carcasses were then cut into four sections (leg cuts, rack, shoulder and loin) and the weight of each component was recorded. The linear dimensions of the refrigerated carcasses and *longissimus* muscle that were measured were rib fat depth (J), tissue depth (GR), eye muscle width (A), eye muscle area, eye muscle depth (B), and fat depth (C). The meat quality parameters of the *longissimus* muscle that were measured were water holding capacity (WHC), shear force, pH, color (CIE L*a*b* coordinates) and cooking loss using procedures shown in Obeidat et al. [10]. 

### Statistical Methods

By using mixed procedures of SAS (Version 8.1, 2000, SAS Inst. Inc., Cary, NC, USA) [11], where the fixed effect in the statistical model was the diet and the animal is nested to diet as the residual error, data were exposed to analysis of variance. By using pair-wise t-tests, mean was separated for all traits. The significance was considered at *p* < 0.05.

## 3. Results

The chemical composition of the DDGS was 273, 272, 104 and 43 g/kg DM for CP, neutral detergent fiber (NDF), acid detergent fiber (ADF) and ether extract (EE) respectively. This ensured that including the DDGS in the diets met their nutritional requirements. The chemical composition of the diets did not change by the inclusion of DDGS. However, the cost of DDGS-containing diets decreased by 5 and 10% for the DDGS125 and DDGS250 respectively, compared with the CON diet (Table 1). 

Intake of DM, CP, ADF and NDF did not differ (*p* > 0.05) among diet groups (Table 2). However, intake of EE was greater (*p* < 0.05) for the DDGS250 diet group than for the CON and DDGS125 groups. Initial BW, final BW, total gain, ADG and FCR were similar (*p* > 0.05) among all dietary treatment groups.

Dry matter, CP, NDF and ADF digestibility was similar (*p* > 0.05) among diet groups (Table 3). Ether extract digestibility increased (*p* < 0.05) in DDGS250 group than in the CON and DDGS125 groups. Nitrogen intake, N lost in feces and N retention was not affected (*p* > 0.05) by the inclusion of DDGS in the diets. However, N lost in urine was greater (*p* < 0.05) in the CON group than in the DDG125 diet groups, whereas DDGS250 was similar to other two groups.

Fasting BW, carcass cuts weight, hot and cold carcass weight, fat tail, dressing percentage and dissected leg carcass cut weights and percentages were comparable among diets (*p* > 0.05; Table 4). Non-carcass components and leg weight increased (*p* < 0.05) in the DDGS250 diet group than in the DDGS125 diet group, whereas lambs fed the CON diet did not differ from those fed the other two treatment diets in terms of these characteristics.

Similar results were detected among diet groups in terms of leg fat depth, rib fat depth, tissue depth, shoulder fat depth and eye muscle width (Table 5). However, eye muscle depth decreased (*p* < 0.05) in the DDGS250 diet group more than in the CON and DDGS125 diet groups. Eye muscle area and fat depth decreased (*p* < 0.05) in the DDGS125 group than in the DDGS250 and CON diet groups. 

Water holding capacity and meat pH was comparable among diet groups (Table 6). However, cooking loss was greater (*p* < 0.05) in the DDGS125 and DDGS250 groups than for the CON group. Shear force, redness (a*) of *longissimus* muscle and color coordinates increased (*p* < 0.05) in the DDGS250 diet group than in DDGS125 and CON diet groups.

## 4. Discussion 

In response to rising feed prices, DDGS is commonly used as a substitute for conventional feeds in lambs’ and ewes’ diets [1,2]. Corn DDGS is inexpensive compared with conventional feeds [1]. Corn DDGS can play a vital role in formulate inexpensive diets for growing Awassi lambs. Consequently, as the amount of DDGS used in feed is increased, the cost of the feed is reduced. Previous studies conducted in our laboratory have produced similar results in studies where conventional feeds were replaced by alternative feeds, indicating the beneficial use of DDGS in ruminant diets [1,6,12]. 

In this study, DDGS was included in the feed at levels of 0, 125, 250 g/kg DM, replacing part of barley grain (565, 495 and 425 g/kg DM) and soybean meal (165, 110 and 55 g/kg DM). The concentration of the EE in the lambs’ diets increased more in feeds containing corn DDGS than in the CON diet group. This is due to the fact that the content of the EE in the DDGS was higher than the content of the EE in the other ingredients. Although the increase in fat content may impact ruminal fermentation and microbial activities and utilization [13,14], the use of DDGS at 125 or 250 g/kg DM did not affect DM and other nutrient intakes.

In this study, including DDGS at levels of 0, 125 and 250 g/kg had no effects on DM, CP, NDF or ADF intakes in the diets of growing Awassi lambs. The influences of inclusion of DDGS in the diets of ruminants were not consistent with results of existing studies. Tanaka et al. [15] and Westreicher-Kristen et al. [16] reported similar DM intake when DDGS was fed to dairy cows at levels of more than 200 g/kg of dietary DM or at levels of 100 or 150 g/kg DM, as reported by Leonardi et al. [17], when fed to lambs at levels of 75 and 150 g/kg DM [2] or when fed to ewes at 200 and 300 g/kg DM [1]. Conversely, Janicek et al. [18] reported an increase in DM intake when dairy cows where fed DDGS at levels of 0, 100, 200, and 300 g/kg DM. A decline in DM intake was reported when DDGS was fed at 310 g/kg DM [19] or at 200 g/kg DM [20]. Differences among studies in DM and other nutrient intakes due to the inclusion of DDGS could partially be related to variations in the inclusion rate of DDGS and the other ingredients of the diets.

Results relating to BW, ADG and FCR were concurrent with previous studies that were conducted in our laboratory [21,22], indicating that the diets we used did not influence the palatability due to the inclusion of DDGS and, in turn, their BW changes. Similarly, growth performance was not affected when DDGS was fed at 230 g/kg for beef cattle or at 75 and 150 g/kg DM for lambs [1,23]. However, previous studies have shown that ADG and feed efficiency enhanced when DDGS was included in beef cattle feed at different levels [23,24].

Feeding DDGS at 125 or 250 g/kg DM did not influence the digestibility of CP, NDF and ADF. In accordance with our result, Winterholler et al. [25] found that NDF, ADF and CP digestibility was similar in beef cows fed three different level of DDGS treatments (0.77, 1.54 or 2.31 kg/d). Conversely, Felix et al. [26] found that DM digestibility reduced linearly when the DDGS fed at 200, 400 or 600 g/kg DM in lamb rations. One possible explanation for this phenomenon could be that the level of EE in the diet was over the threshold, which, in turn, prevented the microbes from attaching to the rumen digesta and blocked the enzyme from digesting the fiber. The fat that is present in the DDGS is mainly composed of unsaturated fatty acid in DDGS, which explains the improvement in the digestibility of EE in the diets having DDGS. 

Awassi lambs whose feed contained DDGS at 125 g/kg and CON exhibited similar amounts of N in feces to lambs receiving the DDGS250 g/kg diet. Lambs receiving the CON diet had a greater amount of urinary N than the DDGS125 g/kg diet group. This is not surprising due to the fact that lambs provided with the CON diet had a higher nitrogen intake and a relatively similar nitrogen digestibility to lambs fed the diets containing DDGS. Felix et al. [26] noted that where the N intake and N digestibility increased, the urinary N output increased which decreased the N retention in lambs fed diets with higher levels of DDGS. However, Gurung et al. [27] found that where the urine N was higher with increasing concentrates containing (0, 12.7, 25.4, 38.1%) of DDGS fed for goats because the DDGS a good sources of RUP. Also, Gurung et al. [27] found that the fecal N, urinary N, absorbed N, and retained N were similar between all treatment which may be there no different on N intake.

Meat quality and carcass traits could be affected by diet ingredients and nutrient contents. The similarities in carcass yields among lambs fed the different diets could be to the similarity in intake, digestibility and N balance that was detected among diet groups. In line with our study, Estrada-Angulo et al. [28] reported no differences between carcass yields in lambs fed DDGS at 250 and 400 g/kg. Abdelrahim et al. [7] reported that carcass characteristics did not affect in lambs fed diets contained DDGS at 127 or 254 g/kg DM. 28.29. Schauer et al. [29] and Huls et al. [30] reported that feeding DDGS for lambs at 600 g/kg and 229 g/kg, respectively, DM did not change the carcass characteristics. In the present study, eye muscle area and fat depth decreased more in the DDGS125 diet group compared with the DDGS250 and CON diets groups. This decrease in eye muscle area could be due to the numerical differences in final BW among diets. Although lambs fed the DDGS250 diet showed greater shear force, all values of meat quality were within the acceptable value. Previous studies showed that feeding DDGS at different levels did not impact upon the shear force [31,32]. This study of DDGS-based diets has shown that there is little effect on carcass meat quality and characteristics by using such feeds. This highlights the potential for using DDGS as an alternative feedstuff without causing any adverse effect on carcass characteristics traits. With regards the color coordinates, Obeidat et al. [10] reported similar results with Awassi lambs slaughtered at the same age but different diets. However, Polidori et al. [33] reported different color values of Fabrianese lambs indicating that the differences may be due to the breed differences. Shear force values were comparable with results obtained in previous studies for lambs slaughtered at the same age [10,33]. Similar to our results, Fleix et al. [26] observed that whiteness did not change when lambs fed DDGS at 0 or 200 g/kg, whereas; it changed at 400 or 600 g/kg DM.

## 5. Conclusions

The results of the present study regarding growing Awassi lambs show that inclusion of DDGS at 125 and 250 g/kg DM did not have a negative effect on intake, digestibility, meat quality and carcass characteristics. However, the cost of feed is reduced indicating that the usage of DDGS in diets for ruminants would be highly beneficial for both producers and consumers. 

## Figures and Tables

**Table 1:** Ingredients and chemical composition of diets fed to growing Awassi lambs containing increasing concentrations of dried distillers’ grains with solubles (DDGS), replacing barley grain, soybean meal, and wheat straw.

Item	Diet ^1^		
	CON	DDGS125	DDGS250
Ingredients (g/kg DM)			
Barley	56.5	49.5	42.5
Soybean meal	16.5	11.0	5.5
Wheat straw	25.0	25.0	25.0
DDGS	0.0	12.5	25.0
Salt	1	1	1
Limestone	1	1	1
Vitamin-mineral premix ^2^	+	+	+
Cost (US$/Ton)	359	341	324
Nutrients (g/kg DM)			
Dry matter	91.05	90.9	90.5
Crude protein	15.99	15.96	15.93
Neutral detergent fiber	28.39	30.00	31.62
Acid detergent fiber	18.07	18.32	18.57
Ether extract	1.91	2.20	2.49

^1^ Diets were (1) no DDGS (CON), (2) 125 g/kg DDGS (DDGS125), and (3) 250 g/kg DDGS (DDGS250). ^2^ Composition per kg use (vitamin A, 600,000 IU; vitamin D3, 200,000 IU; vitamin E, 75 mg, vitamin K3, 200 mg; vitamin B1, 100 mg; vitamin B5, 500 mg; lysine 0.5%; DL-methionine, 0.15%; manganese oxide, 4000 mg; ferrous sulphate, 15,000 mg; zinc oxide, 7000; magnesium oxide, 4000 mg; potassium iodide, 80 mg; sodium selenite, 150 mg; copper sulphate, 100 mg; cobalt phosphate, 50 mg, dicalcium phosphate, 10,000 mg).

**Table 2:** Effects of feeding dried distillers’ grains with solubles (DDGS) on nutrient intakes and growth performance of growing Awassi lambs.

Item	Diet ^1^			
	CON	DDGS125	DDGS250	SE
Nutrient intake, g/d				
Dry matter	1085	1038	1118	63.3
Crude protein	174	166	178	10.1
Neutral detergent fiber	308	311	353	19.0
Acid detergent fiber	196	190	208	11.6
Ether extract	21 ^a^	23 ^a^	28 ^b^	1.4
Initial weight, kg	15.3	15.0	15.6	0.99
Final weight, kg	33.3	31.8	35.2	1.18
Total gain, kg	18.0	16.8	19.6	0.99
Average daily gain, g/d	214	199	233	11.77
FCR ^2^	5.1	5.5	4.8	0.40

^1^ Diets were (1) no DDGS (CON), (2) 125 g/kg DDGS (DDGS125), and (3) 250 g/kg DDGS (DDGS250), ^2^ FCR: Feed conversion ratio. ^a,b^ Means within a row without a common superscript differ significantly (*p* < 0.05).

**Table 3:** Effects of feeding dried distillers’ grains with solubles (DDGS) on nutrient digestibility, N balance and rumen fluid pH of growing Awassi lambs.

Item	Diets ^1^			
	CON	DDGS125	DDGS250	SE
Digestibility, %				
Dry matter	76.4	71.7	72.4	2.65
Crude protein	76.0	73.5	73.0	2.17
Neutral detergent fiber	63.8	56..4	61.2	4.43
Acid detergent fiber	55.0	47.3	51.1	5.15
Ether extract	62.7 ^a^	67.2 ^ab^	72.0 ^b^	3.50
N balance				
N intake, g/d	30.2	25.0	27.3	2.27
N in feces, g/d	7.3	6.3	7.3	0.70
N in urine, g/d	6.4 ^a^	4.4 ^b^	2.3 ^c^	0.62
N retained, g/d	16.5	14.3	17.7	1.84
Retention, g/100 g	54.6	57.2	64.8	4.80

^1^ Diets were (1) no DDGS (CON), (2) 125 g/kg DDGS (DDGS125), and (3) 250 g/kg DDGS (DDGS250). ^a,b^ Within a row, means without common superscripts differ (*p* < 0.05).

**Table 4:** Effects of feeding dried distillers’ grains with solubles (DDGS) on carcass, non-carcass components, dissected leg carcass cut weights and percentages of growing Awassi lambs.

Item	Diets ^1^			
	CON *n* = 5	DDGS125 *n* = 5	DDGS250 *n* = 5	SEM
Fasting live weight (kg)	31.3	30.2	33.2	1.43
Hot carcass weight (kg)	14.8	14.4	16.1	0.75
Cold carcass weight (kg)	14.5	14.0	15.8	0.75
Dressing percentage	46.4	46.1	47.6	0.72
Non-carcass components (kg) ^2^	1.32 ^b^	1.24 ^a^	1.40 ^b^	0.05
Carcass cut weights (kg) ^3^	13.1	12.5	14.2	0.57
Fat tail (kg)	1.03	1.23	1.40	0.14
Leg weight (kg)	2.60 ^ab^	2.41 ^a^	2.83 ^b^	0.97
Subcutaneous fat (g/100 g)	12.0	12.6	11.1	1.2
Intermuscular fat (g/100 g)	2.4	2.5	2.4	0.17
Total fat (g/100 g)	14.5	15.1	13.5	1.18
Total meat (g/100 g)	55.2	57.7	57.9	1.10
Total bone (g/100 g)	20.7	20.9	22.4	0.76
Meat to bone ratio	2.71	2.80	2.62	0.12
Meat to fat ratio	3.90	4.11	4.52	0.43

^1^ Diets were (1) no DDGS (CON), (2) 125 g/kg DDGS (DDGS125), and (3) 250 g/kg DDGS (DDGS250), ^2^ Non-carcass components (Heart, liver, spleen, kidney, and lungs and trachea), ^3^ Carcass cut (shoulder, racks, loins, and legs). ^a,b^ Within a row, means without common superscripts differ (*p* < 0.05).

**Table 5:** Effects of feeding dried distillers’ grains with solubles (DDGS) on carcass leaner dimensions of growing Awassi lambs.

Item	Diets ^1^			
	CON *n* = 5	DDGS125 *n* = 5	DDGS250 *n* = 5	SEM
Leg fat depth (*L3*) (mm)	4.50	3.81	3.51	0.391
Tissue depth (*GR*) (mm)	12.2	12.0	13.5	0.66
Rib fat depth (*J*) (mm)	4.51	4.01	4.32	0.48
Eye muscle width (*A*) (mm)	56.0	53.2	56.2	1.08
Eye muscle depth (*B*) (mm)	25.2 ^b^	25.0 ^b^	22.8 ^a^	0.24
Eye muscle area (cm2)	11.8 ^b^	10.1 ^a^	12.4 ^b^	0.46
Fat depth (*C*) (mm)	3.00 ^b^	2.31 ^a^	2.81 ^b^	0.12
Shoulder fat depth (*S2*) (mm)	3.72	4.01	3.52	0.38

^1^ Diets were (1) no DDGS (CON), (2) 125 g/kg DDGS (DDGS125), and (3) 250 g/kg DDGS (DDGS250). ^a,b^ Within a row, means without common superscripts differ (*p* < 0.05).

**Table 6:** Effects of feeding dried distillers’ grains with solubles (DDGS) on meat quality characteristics of growing Awassi lambs.

Item	Diets ^1^			
	CON *n* = 5	DDGS125 *n* = 5	DDGS250 *n* = 5	SE
pH ^2^	5.80	5.81	5.80	0.01
Cooking loss (g/100 g)	58.1 ^a^	60.4 ^b^	60.8 ^b^	0.66
Water holding capacity (g/100 g)	28.0	27.4	28.3	0.75
Shear force (kg/cm^2^)	5.62 ^a^	5.42 ^a^	6.43 ^b^	0.22
Color coordinates				
L* (whiteness)	37.8	37.5	36.9	0.39
a* (redness)	2.51 ^a^	2.60 ^a^	2.91 ^b^	0.07
b* (yellowness)	19.7	18.9	17.6	0.45

^1^ Diets were (1) no DDGS (CON), (2) 125 g/kg DDGS (DDGS125), and (3) 250 g/kg DDGS (DDGS250), ^2^ pH measured after thawing. ^a,b^ Within a row, means without common superscripts differ (*p* < 0.05).
